# Sensitivity to near-future CO_2_ conditions in marine crabs depends on their compensatory capacities for salinity change

**DOI:** 10.1038/s41598-018-34089-0

**Published:** 2018-10-23

**Authors:** Nia M. Whiteley, Coleen C. Suckling, Benjamin J. Ciotti, James Brown, Ian D. McCarthy, Luis Gimenez, Chris Hauton

**Affiliations:** 1 School of Natural Sciences, Bangor University, Deiniol Road, Bangor, Gwynedd LL57 2UW UK; 2 School of Ocean Sciences, Bangor University, Askew Street, Menai Bridge, Anglesey, LL59 5AB UK; 3 Ocean and Earth Science, University of Southampton, Waterfront Campus, National Oceanography Centre Southampton, European Way, Southampton, SO14 3ZH UK; 4 Present Address: University of Rhode Island, Fisheries, Animal and Veterinary Sciences, Kingston, Rhode Island 02881 USA; 5 Present Address: School of Biological and Marine Sciences, University of Plymouth, Drake Circus, Plymouth, PL4 8AA UK

## Abstract

Marine crabs inhabit shallow coastal/estuarine habitats particularly sensitive to climate change, and yet we know very little about the diversity of their responses to environmental change. We report the effects of a rarely studied, but increasingly prevalent, combination of environmental factors, that of near-future *p*CO_2_ (~1000 µatm) and a physiologically relevant 20% reduction in salinity. We focused on two crab species with differing abilities to cope with natural salinity change, and revealed via physiological and molecular studies that salinity had an overriding effect on ion exchange in the osmoregulating shore crab, *Carcinus maenas*. This species was unaffected by elevated CO_2_, and was able to hyper-osmoregulate and maintain haemolymph pH homeostasis for at least one year. By contrast, the commercially important edible crab, *Cancer pagurus*, an osmoconformer, had limited ion-transporting capacities, which were unresponsive to dilute seawater. Elevated CO_2_ disrupted haemolymph pH homeostasis, but there was some respite in dilute seawater due to a salinity-induced metabolic alkalosis (increase in HCO_3_^−^ at constant *p*CO_2_). Ultimately, *Cancer pagurus* was poorly equipped to compensate for change, and exposures were limited to 9 months. Failure to understand the full spectrum of species-related vulnerabilities could lead to erroneous predictions of the impacts of a changing marine climate.

## Introduction

Estuarine and shallow coastal habitats are complex ecosystems of high productivity facing conflicting socio-economic and environmental demands^1^. Estuaries, for instance, are important areas for fisheries and aquaculture production and yet are challenging environments in which to live because of the co-occurrence of fluctuations in salinity, temperature, pH and oxygen on different spatial and temporal scales^2^. Climate change is inflicting further environmental change with global increases in atmospheric CO_2_ leading to elevated sea surface temperatures and CO_2_-driven reductions in ocean pH, broadly termed ‘ocean acidification’ (OA)^3^. Increased surface temperature is also leading to an intensification of the global water cycle resulting in changing patterns of ocean surface salinity^4^. Shallow coastal and estuarine regions may experience greater changes in pH and salinity than the open ocean through an increased frequency of exceptional storm events and freshwater runoff from terrestrial flooding^5,6^.

The impacts of reductions in salinity and pH on marine communities and populations are difficult to assess because of the complexity of species and community interactions, and the time scales involved in observing changes in biodiversity, abundance and geographical range^7^. Individual physiologies, however, are ecologically relevant as they may provide mechanistic explanations for differences in sensitivity, performance, adaptive potential, and ultimately survival to environmental change among species and taxa^8,9^. For example, physiological responses to salinity change influence salinity tolerances of aquatic organisms and can be related to community structure and boundaries of species distribution^2^. Indeed, marine invertebrates demonstrate a range of osmoregulatory strategies from independent regulation of body fluid osmolality to conformity^10^. Conventionally, species that demonstrate active life styles with high metabolic rates are considered less vulnerable^7,11–13^. Physiologically, it is argued that such species have greater capacities for ion exchange and for buffering and transporting CO_2_ in the blood^8,11,12,14^. However, much of our understanding of these responses comes from short-term experiments (e.g. days to weeks) and acute exposures to single stressors^12,14,15^. Of more concern, comparatively little is known about physiological responses to environmentally relevant changes under more realistic climate change scenarios of multiple interacting environmental drivers over the longer term (months to years)^16–19^.

Multifactorial experiments are beginning to show interactive effects on an organism’s physiology, which might have significant longer-term repercussions on performance than each factor in isolation^19–23^. The research focus to date, however, has been on elevated *p*CO_2_ and warming^20,24^. Experiments investigating combined effects of elevated *p*CO_2_ and salinity have received much less attention, especially the ability of species to compensate for CO_2_-induced changes in extracellular pH (acid-base status). Compensation of extracellular pH is fundamental to survival during elevated *p*CO_2_^12,13,15,25^, but is itself influenced by salinity, with seawater dilution disrupting extracellular acid-base status^26^ and energy status^27^. The combined effects of both environmental factors are difficult to predict as acid-base adjustments occur via ion exchange mechanisms, which may also have the opposing function of ion uptake during low salinity exposure for the purposes of osmoregulation. Ion regulation, however, is an energetically demanding process suggesting that osmoregulation in marine invertebrates under low salinity may be a distinct disadvantage in the longer-term due to trade-offs with ecologically important processes such as growth and reproduction^15,27,28^.

Marine crabs are important predators of molluscs, polychaetes and other crustaceans and have significant effects on community structure in shallow coastal and estuarine ecosystems. Many crab species are also commercially important and increasingly contribute to global food security through capture fisheries and aquaculture. Marine crustaceans are generally considered to be more tolerant of increasing CO_2_ levels than other taxa, but physiological studies are mainly limited to those species more capable of coping with environmental change^12,13^. Here we test these broad assumptions about the performance of key taxa, and explore the prediction that compensatory capacities are complex, highly variable but intrinsically linked with the ability to ion and osmo-regulate^12^ by examining two species of marine crabs with differing abilities to compensate for salinity change. We studied a moderate osmoregulator, the intertidal/estuarine shore crab, *Carcinus maenas* and an osmoconformer, the subtidal edible crab, *Cancer pagurus. Carcinus maenas* is euryhaline and capable of hyper-osmoregulation down to an external salinity of 8^29^, but *Cancer pagurus* is stenohaline, and prefers marine habitats usually buffered from salinity change. *Carcinus maenas* demonstrates remarkable environmental tolerance, has a wide geographical distribution and is highly invasive outside of Europe^30^. *Cancer pagurus* inhabits shallow shelf waters of the NE Atlantic from tidal levels (juveniles) down to 50–100 m (adults)^31^, and supports one of the most important fisheries in Europe^32^. Little is known about *Cancer pagurus* physiology, apart from the fact that it is an osmoconformer unable to maintain body fluid osmolality separate from that of the external environment^33^, and is more sensitive to rising temperatures when exposed to high CO_2_^34^. We exposed both species to combinations of *p*CO_2_ (ambient at 400 µatm vs ‘business as usual’ predictions for 2100 at ~1000 µatm), and salinity (SW, full strength salinity = 33 vs DW, reduced salinity = 25) according to a fully factorial design, and studied key physiological processes of pH homeostasis and ion regulation for up to 12 months. The purpose here was to provide a physiological framework to explain species-specific differences in CO_2_ tolerances, to further understand vulnerabilities of marine invertebrates to future coastal/estuarine environments.

## Results

### Seawater Chemistry

Seawater locally sourced from the Menai Strait was unpolluted and had an ionic composition in agreement with that of reference seawater (Table 1 in^35^). Variations in seawater carbonate chemistry were greater among treatments than within treatments over time. The seawater manipulations produced reasonably stable and accurate experimental parameters as detailed in Supplementary Table S1. Overall, salinity had no effect on seawater *p*CO_2_ but A_T_ and DIC were significantly higher and pH significantly lower in SW compared with DW. Under elevated *p*CO_2_, seawater *p*CO_2_ and DIC increased but A_T_ was unaffected (Supplementary Table S1). Temperature varied over time in all four treatments, but all treatments showed the same temporal pattern.

### Haemolymph Acid-base Status

**Figure 1:**
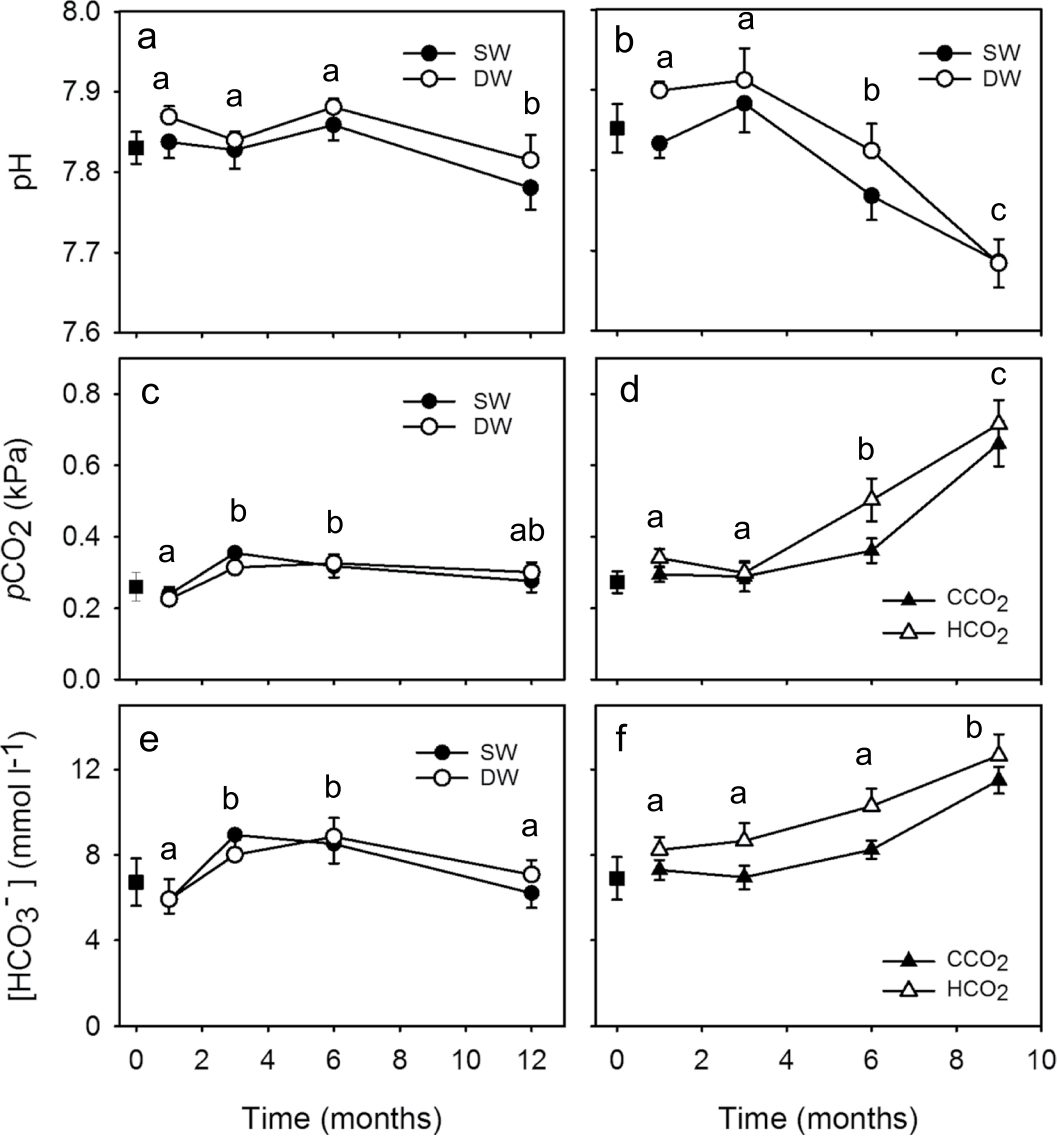
Haemolymph acid-base changes against time in *Carcinus maenas*: (**a**) haemolymph pH; (**c**) partial pressure of CO_2_ (*p*CO_2_); (**e**) bicarbonate concentration ([HCO_3_^−^]). Acid-base changes in *Cancer pagurus*: (**b**) haemolymph pH according to salinity and time (SW = full strength seawater, closed circles; DW = dilute seawater, open circles); (**d**) haemolymph *p*CO_2_ and (**f**) haemolymph [HCO_3_^−^] according to external *p*CO_2_ and time (CCO_2_ = control, closed triangles; HCO_2_ = elevated *p*CO_2_, open triangles). Values given as means ± SEM. For all acid-base variables in *Carcinus maenas n* = 27, 25, 30 and 24 at 1, 3, 6 and 12 months, respectively. For haemolymph pH in *Cancer pagurus*: *n* = 15 at 1 month and *n* = 16 at 3, 6 and 12 months in SW and DW, apart from 9 months in DW when *n* = 12. For haemolymph *p*CO_2_ and [HCO_3_^−^] in *Cancer pagurus*: *n* = 15 at 1, 3 and 9 months at CCO_2_ and HCO_2_, apart from 9 months in HCO_2_ when *n* = 13, and *n* = 16 at 6 months in both conditions. Values with different letters show significant differences (SNK *post hoc*, *P* < 0.05). In 1a, c and e, both SW and DW shown for reference even though salinity had no effect (significant differences related to changes over time). All values in control crabs at one month (CCO_2_, SW) were similar to those measured in baseline crabs (closed squares; t-tests, df = 13, *P* = 0.457 to 0.996).

To determine the ability of crabs to maintain extracellular acid-base homeostasis, we measured haemolymph pH, partial pressure of CO_2_ (*p*CO_2_), and bicarbonate concentration ([HCO_3_^−^]). In *Carcinus maenas*, all three acid-base parameters varied with time (Fig. 1a,c,e; Table 1), but were unaffected by either salinity or *p*CO_2_ (Supplementary Tables S3 and S4). Haemolymph pH remained unchanged from one to 6 months, but fell significantly between 6 and 12 months (Fig. 1a). Haemolymph *p*CO_2_ and [HCO_3_^−^] were significantly higher at 3 and 6 months than at one month, but declined again at 12 months (Fig. 1c,e).

**Table 1:** Summary of the models that best explain variation in the physiological parameters for both crab species.

Species	Physiological parameter	Terms included in best model	
		Variance structure	Fixed Effects
*Carcinus maenas*	Haemolymph pH	Sal:*p*CO_2_	Time
	Haemolymph *p*CO_2_	None	Time
	Haemolymph [HCO_3_^−^]	None	Time
	Osmolality	None	Sal + Time
	Gill NKA activity	None	Sal
*Cancer pagurus*	Haemolymph pH	Time	Sal + Time
	Haemolymph *p*CO_2_	Sal:Time	*p*CO_2_ + Time + CW
	Haemolymph [HCO_3_^−^]	*p*CO_2_	*p*CO_2_ + Time + CW
	Osmolality	Sal:Time	Additive + *p*CO_2_:CW + Time:CW
	Gill NKA activity	None	CW

NKA = Na^+^/K^+^ ATPase activities in posterior gill 8.

Models represent simplifications of a global model including four fully crossed variables (seawater *p*CO_2_; salinity (Sal); and time as fixed factors; body size (CW) as a covariate). An error variance structure is included crossing *p*CO_2_, salinity and time.

Models were selected using AICc and log-likelihood ratio tests as detailed in Methods, and in Supplementary Tables S3 –S6.

All four explanatory variables (CO_2_, salinity, time, body size) influenced haemolymph acid-base status in *Cancer pagurus* but in different combinations depending on acid-base parameter. Haemolymph pH varied according to salinity and time, but the salinity effect was consistent over the course of the experiment (Fig. 1b; Tables 1, S5 and S6). Haemolymph pH was significantly lower in SW vs DW crabs at 7.79 ± 0.02 (*n* = 63) and 7.83 ± 0.02 (*n* = 59), respectively (Supplementary Table S6). Haemolymph *p*CO_2_ and [HCO_3_^−^] were higher under elevated seawater *p*CO_2_, irrespective of the significant effect of time (Tables 1 and S6; Fig. 1d,f), and the small but significant increase in both acid-base variables with increase in body size (Supplementary Figs S1 and S2; Table S6). For example, mean haemolymph *p*CO_2_ in *Carcinus maenas* at 6 months was 0.36 ± 0.03 µatm in ambient and 0.50 ± 0.06 µatm in elevated *p*CO_2_ (Fig. 1d). In the same month, [HCO_3_^−^] was 8.25 ± 0.43 mmol L^−1^ in ambient but 10.29 ± 0.82 mmol L^−1^ in elevated *p*CO_2_ (Fig. 1f). In all cases *n* = 16.

Haemolymph osmolality was determined to assess the ability of the crabs to osmoregulate.

In *Carcinus maenas*, haemolymph osmolality varied due to salinity and time (Tables 1 and S3), but *p*CO_2_ had no effect. Haemolymph osmolality was significantly lower in DW vs SW crabs at 839 ± 14 (*n* = 50) and 944 ± 10 (*n* = 51) mosmol kg^−1^, respectively (Supplementary Table S4). Over time, osmolality fell significantly at 3 months (SNK *post hoc P* < 0.05), but values recovered after 6 and 12 months exposure. Haemolymph osmolality in DW crabs remained 103 to 175 mosmol kg^−1^ above that of external DW, apart from 3 months when haemolymph osmolality was only 44 mosmol kg^−1^ above that of DW.

The main effect on haemolymph osmolality in *Cancer pagurus*, was salinity, but there were also interactive effects of body size with CO_2_, and with time (Tables 1 and S5). As the effects of CO_2_ and body size were relatively small (Supplementary Fig. S3), the most important factors were salinity and time. Haemolymph osmolality was significantly lower in DW vs SW crabs at 757 ± 9 (*n* = 59) and 947 ± 8 (*n* = 62) mosmol kg^−1^, respectively (Supplementary Table S5), but remained 22 mosmol kg^−1^ above that of the dilute seawater in DW crabs.

Branchial NKA activities were examined in posterior gill 8 to determine whether crabs were engaging active, energy consuming mechanisms to maintain acid-base status and osmoregulation. Salinity had a highly significant effect on NKA activities in *Carcinus maenas* (Tables 1 and S4) with activities 1.5 fold higher in DW vs SW crabs at 3.86 ± 0.13 (*n* = 54) and 2.5 ± 0.08 (*n* = 51) µmol ADP mg^−1^ protein h^−1^, respectively. By contrast, elevated *p*CO_2_ had no effect on NKA activity in *Carcinus maenas*. NKA activities in the posterior gill 8 of *Cancer pagurus* were unaffected by any of the explanatory variables (Tables 2 and S5), apart from a small increase in NKA activities with increase in body size, which was marginally significant (Table S6). Mean NKA activities in *Cancer pagurus* gills were 1.07 ± 0.03 (*n* = 110) µmol ADP mg^−1^ protein h^−1^.

**Table 2:** Summary of models that best explain variation in expression of six genes in the posterior gills of both crab species.

Species	Gene	Gene category	Terms included in best model:	
			Variance structure	Fixed effects
*Carcinus maenas*	*AE*	Ion and AB	Time	Time + CW + Time:CW
	*CAc*	Ion	None	Additive + all 2-way interactions + *p*CO_2_:Time:CW + Sal:Time:CW
	*gpi-CA*	Respiration	None	Sal
	*NKAα*	Ion and AB	None	Sal
	*NHE*	Ion and AB	None	Sal + Time + CW + Sal:CW + Time:CW
	*VATB*	Ion and AB	None	Sal
*Cancer* *Pagurus*	*AE*	Ion and AB	None	None
	*CAc*	Ion	None	Time
	*gpi-CA*	Respiration	None	None
	*NKAα*	Ion and AB	None	None
	*NHE*	Ion and AB	na	na
	*VATB*	Ion and AB	None	Sal + Time + CW

*AE* = anion exchanger protein, *CAc* = cytoplasmic carbonic anhydrase, *gpi-CA* = gpi-linked carbonic anhydrase, *NKAα* = Na^+^/K^+^ ATPase alpha subunit, *NHE* = Na^+^/H^+^ exchanger, and *VATB* = vacuolar ATP synthase subunit B. AB = acid base.

Models represent simplifications (where possible) of a global model including four fully crossed explanatory variables as explained in Table 1 and an error variance structure crossing *p*CO_2_, salinity and time.

Model terms were selected using AIC_c_ and log-likelihood ratio tests as detailed in Methods and in Supplementary Tables S7 and S8.

### Branchial gene expression

Gene transcription of several ion transporting genes were quantified to examine the underlying mechanisms responsible for ion and acid-base regulation in the posterior gills of marine crabs. We measured the ion transporting enzymes: Na^+^/K^+^-ATPase α-subunit, *NKAα*; cytoplasmic carbonic anhydrase, *CAc*; and gpi-linked carbonic anhydrase, *gpi*-*CA*. We also examined the ion exchangers: anion exchanger protein (Cl^−^/HCO_3_^−^), *AE*; sodium/proton exchanger, *NHE*; and V-type H^+^ ATPase B-subunit, *VATB*. Justification for the choice of genes is given in Methods.

**Figure 2:**
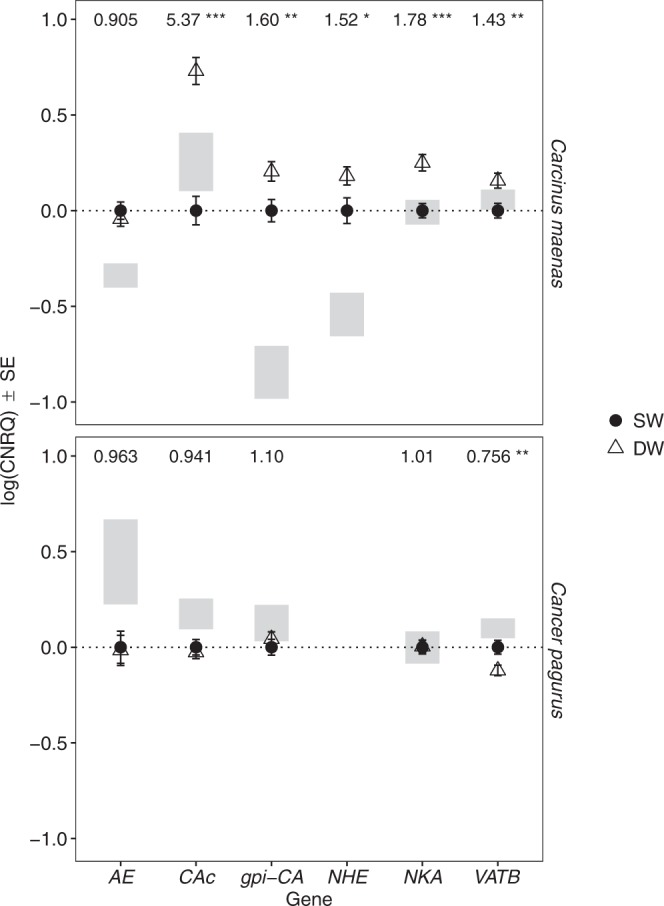
Expression of selected genes as a function of salinity. Gene expression under control (SW, closed circles) and dilute seawater (DW, open triangles) salinity across all time points and *p*CO_2_ treatments. Gene expression values scaled relative to values in the control salinity treatment. Numbers at the top of the plots represent the proportional change in expression between control and low salinity. Asterisks represent the significance of these difference from t-tests, where *P* = 0.05–0.01 (*), 0.01–0.001 (**) and <0.001 (***). Shaded areas represent the spread (SE) of expression values at the end of acclimation period and prior to the start of the experiment. For *Carcinus maenas*, *n* = 62 for SW and for DW crabs. For *Cancer pagurus*, *n* = 64 for SW, and *n* = 61 for DW.

**Figure 3:**
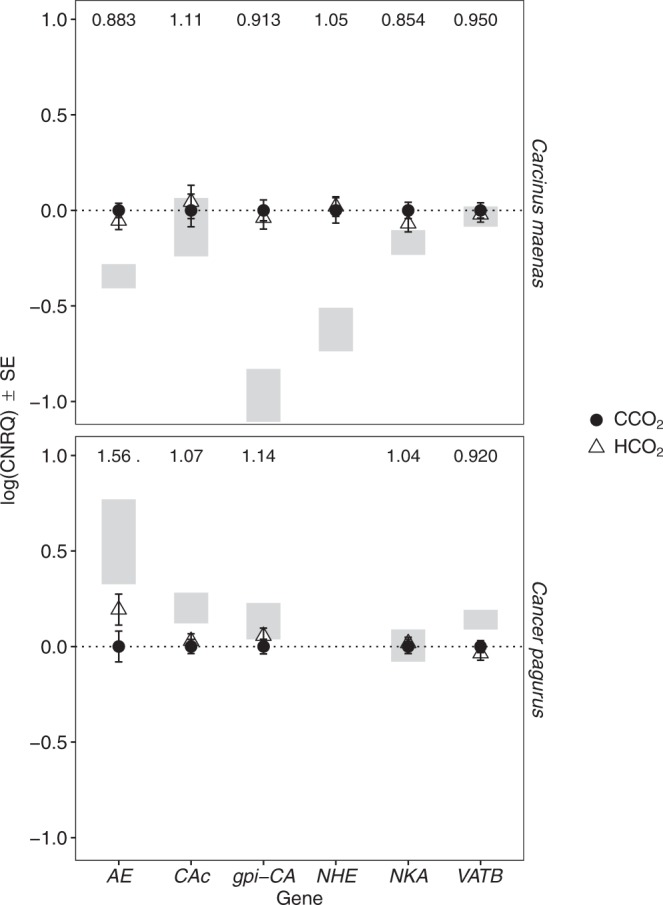
Expression of selected genes as a function of *p*CO_2_ level. Gene expression under control (closed circles) and elevated (open triangles) *p*CO_2_ across all time points and salinity treatments. Gene expression values scaled relative to values in the control *p*CO_2_ treatment. Numbers at the top of the plots represent the proportional change in expression between control and elevated *p*CO_2_. Explanation for asterisks and shaded areas given in Fig. 2. For *Carcinus maenas*, *n* = 62 for both CCO_2_ and HCO_2_. For *Cancer pagurus*, *n* = 64 for CCO_2_, and *n* = 61 for HCO_2_.

In *Carcinus maenas*, *NKAα* expression in gill 8 varied due to a relatively weak 3-way interaction between salinity, *p*CO_2_ and body size (Supplementary Table S7 and Fig. S4). Further analysis of the simpler models demonstrated that salinity had an important effect with significantly higher values in DW crabs (Table 2; Fig. 2). Variation in *CAc* was influenced by 3-way interactions between time and body size, and either salinity or *p*CO_2_ (Supplementary Fig. S5; Tables 2 and S7). Further analysis revealed that there was a clear effect of salinity, regardless of time and body size, with higher *CAc* expression in DW crabs (Fig. 2). In contrast, elevated *p*CO_2_ had no effect (Fig. 3). Salinity was the only factor to influence *gpi-CA* and *VATB* gene expression in *Carcinus maenas* (Tables 2 and S7). Both genes were upregulated in DW but to a lesser extent than *CAc* (Fig. 2). *NHE* expression for *Carcinus maenas* varied due to a 3-way interaction between *p*CO_2_, time and body size (Supplementary Table S7). However, exploration of simpler models revealed an interaction between salinity and body size, as well as time and body size (Tables 2 and S7; Fig. S6). At one and 3 months, *NHE* expression tended to be lower in the smallest SW *Carcinus maenas* (CW < 30 mm at one month and <35 mm at 3 months) (Supplementary Fig. S6). At the same sampling intervals, smaller crabs showed an increase in *NHE* expression in DW. When considered as a main effect, salinity significantly affected *NHE* expression (Fig. 2). *AE* was unaffected by salinity or *p*CO_2_, but instead depended on body size and sampling time (Tables 2 and S7). *AE* expression increased with body size at one and 3 months, but declined with body size at 6 and 9 months (Supplementary Fig. S7).

In contrast to *Carcinus maenas*, *NKAα*, *gpi-CA* and *AE* in gill 8 of *Cancer pagurus* were unaffected by any of the main factors (Tables 2 and S8). *CAc* expression varied with time due to a small down regulation between one and 12 months. *VATB* was the only gene in the gills of *Cancer pagurus* to vary with salinity, and even though *VATB* expression also varied with time and body size, it was consistently lower in DW crabs (Fig. 2).

## Discussion

Here we develop a novel mechanistic understanding of the combined effects of long-term exposure to elevated *p*CO_2_ and reduced salinity in two marine crab species with contrasting ecophysiological characteristics. We provide evidence that physiological responses of crab species with differing experiences of salinity change in their natural environment, affects their ability to cope with elevations in seawater *p*CO_2_. Overall, pH homeostasis in *Carcinus maenas*, the osmoregulator, which has invaded estuarine and intertidal environments, was unaffected by elevated *p*CO_2_ or salinity, either alone or in combination. In marked contrast, the edible crab, the osmoconformer, was unable to maintain pH homeostasis under similar CO_2_ and salinity treatments, although there was some respite to acid-base disruptions in dilute seawater. All DW crabs at a salinity of 25 experienced higher external pH values and lower A_T_ values than their SW counterparts (Supplementary Table S1). Such changes in seawater carbonate chemistry result from salinity-induced changes in dissociation constants for carbonic acid and solubility coefficients for CO_2_^36^. Physico-chemical changes in seawater can influence haemolymph *p*CO_2_ levels in *Carcinus maenas* but are not known to affect the metabolic alkalosis caused by reduced salinity (reviewed in^12^). With this in mind, we discuss below the potential mechanisms underlying the contrasting abilities of these two species to compensate for external changes in CO_2_ and salinity, and consider how this information can generally inform on the vulnerability of osmoregulators vs osmoconformers in a changing ocean.

### Effects of reduced salinity and elevated CO_2_ on the osmoregulator

Haemolymph acid-base status in *Carcinus maenas* was unaffected by either a salinity reduction to 25, or an elevation in *p*CO_2_ to 1000 µatm, or both factors in combination. Minor adjustments in haemolymph [HCO_3_^−^] occurred over time to compensate for small elevations in haemolymph *p*CO_2_, and despite a slight fall in haemolymph pH at 12 months, all 3 acid-base parameters remained well within the normal range of values reported for aquatic crabs^37^. *Carcinus maenas* was clearly capable of fully compensating for the treatment combinations over 12 months exposure. First, because haemolymph *p*CO_2_ was unaffected by the near-future increases in seawater *p*CO_2_ (~1000 µatm, 0.10 kPa), which suggested the maintenance of an outward diffusion gradient for CO_2_ excretion across the gills^11,37^. Second, because external reductions in salinity had no effect on haemolymph pH, which usually increases in marine crabs during short-term salinity transfer through the increased elimination of H^+^ during enhanced Na^+^ uptake^26^. Compensation over time is likely to have involved branchial HCO_3_^−^ uptake from external seawater, which is the principal mechanism for extracellular acid-base regulation in aquatic crabs^37^.

In *Carcinus maenas*, salinity was the only factor to affect NKA activity in posterior gill 8. NKA activities were significantly higher in DW than SW crabs as previously observed in euryhaline crabs, including *Carcinus maenas*, after transfer to low salinity^29,38^. This large, trans-membrane transport enzyme is a critical component of low salinity acclimation in osmoregulating crabs, as it establishes electrochemical gradients across the gill and provides the major driving force for the transepithelial movement of ions^29,39^. During low salinity exposure, increased NKA activities in the posterior gills are associated with the active branchial absorption of Na^+^ and Cl^−^ to compensate for the passive, diffusive loss of these ions. The NKA activities obtained in the posterior gills of SW crabs in this investigation match those obtained by^40^. The moderate 2-fold increase in NKA activities observed in DW crabs, however, was smaller than that previously reported for *Carcinus maenas* during shorter term exposures to lower salinities (3 weeks at a salinity of 10)^41^. The difference here was the maintenance of elevated NKA activities in the posterior gills of DW crabs for 12 months, despite the possible energetic repercussions associated with NKA activity as a major energy-demanding process^42^.

The current gene transcription experiment supports an increasing role for ion regulation in *Carcinus maenas* during dilute seawater exposure. Five out of the 6 genes responsible for both ion and acid-base regulation were upregulated in response to salinity. For *NKAα*; *gpi-CA* and *VATB*, salinity was the main driver resulting in upregulation of all 3 genes. Collectively these responses indicate an increasing dependence on ion regulation during exposure to dilute seawater to compensate for the high rate of ion loss and increased passive uptake of water^43,44^. More specifically, upregulation of *NKAα* in combination with increased NKA activities suggests proliferation of specialised cells for osmo- and ion exchange in the posterior gills, and an increased ability to drive ion exchange across the gill epithelium against a concentration gradient^29,45^. The salinity-induced increase in *VATB* expression may support the movement of Na^+^, K^+^ and Cl^−^ across the apical membrane, and it may also increase the rate at which H^+^ provided by CAc is pumped out of the cytoplasm, assuming the proton pump is located on the apical membrane^38,46,47^. The salinity-induced upregulation of *gpi-CA* is harder to explain as this enzyme is present on the basolateral membrane of gill epithelia and mainly has a respiratory role in enhancing CO_2_ excretion^48^. However, upregulation of *gpi*-*CA* in the posterior gills of *Carcinus maenas* also occurs in response to short-term, low salinity transfer and may be associated with the combined effects of increased metabolic rate (increased rate of CO_2_ excretion) and the proliferation of ion transporting cells in low salinity^48^.

Reduced salinity also caused the upregulation of 2 further genes: *CAc* and *NHE*. Although *p*CO_2_ interacted with salinity and with body size in the present study, further inspection of the relationships revealed that salinity was the main factor responsible for the upregulation of *CAc*. Increased *CAc* expression represents a permanent transcriptional response to low salinity, and indicates an increase in CAc activity^44^. CAc plays a central role in both ion and acid-base regulation as it catalyses the hydration of CO_2_ in the cytoplasm of gill epithelia to provide counter ions (H^+^ and HCO_3_^−^) for both anion and NHE exchange^29,48,49^. Previous studies demonstrate that CAc is directly involved in osmo- and ion regulation in several euryhaline crabs including *Carcinus maenas*^44,50^. Our studies further support this view as the *CAc* gene was the most sensitive to reduced salinity suggesting an important involvement in ion regulation as outlined by^48^. Upregulation of *NHE* in DW crabs suggests enhanced Na^+^ uptake, as NHE is responsible for exchanging two cations (Na^+^ and possibly NH_4_^+^) for one proton across the apical surface of crab gill epithelia^51^. Previous studies have failed to show an upregulation in *NHE* in *Carcinus maenas* gills after 15 days in low salinity^49^, but the present study demonstrates the importance of NHE in making long-term adjustments.

In sharp contrast to salinity, near-future *p*CO_2_ had no effect on posterior gill NKA activities, or on the transcription levels of the genes measured here in *Carcinus maenas*. Similarly, a *p*CO_2_ of 4,000 µatm (0.4 kPa) had no effect on the genes associated with acid-base regulation in *Carcinus maenas* gills after acute exposures of 7 days and 11 weeks^25^, although the experimental crabs used by these authors originated in the Baltic living at salinities of 14–15, and may already have increased capacities for ion exchange. Collectively, these studies illustrate that the capacity to regulate acid-base disturbances under elevated *p*CO_2_ in osmoregulators is not reliant on biochemical or transcriptional control of ion exchange mechanisms in the posterior gill, although the role of anterior gills warrants further study^52^. Indeed other acute studies have revealed an increase in *NKAα* expression and membrane bound *CA* in isolated, perfused gills from *Carcinus maenas* acclimated to low salinity (S = 10) for 7 days and exposed to a *p*CO_2_ of 3,243 µatm (0.32 kPa)^53^, although upregulation of both genes were only observed in the anterior but not the posterior gills.

As body size co-varied with time in *Carcinus maenas*, it was difficult to assess the independent effects of either factor. Our study suggests that body size had some effect on the gene expression ratios of *CAc*, *NHE* and *AE* in posterior gills (Table 2). It is possible that these size-dependent relationships were due to a decline in weight-specific gill surface area with increase in body mass, as observed in the strong osmoregulating crab, *Callinectes sapidus*, and in several freshwater decapod species^33,54^. The positive relationships between body size and gene expression could also be associated with reductions in gill permeability observed with increase in body size^54^. Clearly, the independent effect of body size on gene expression ratios for branchial ion-transporters requires further investigation.

### Effects of reduced salinity and elevated CO_2_ on the osmoconformer

Our study demonstrates that the edible crab, *Cancer pagurus*, was unable to increase ion regulatory capacities during exposure to reduced salinity even after prolonged exposure for 9 months, as branchial NKA activities and gene expression ratios for all genes, apart from *VATB*, were unaffected by salinity. Moreover, NKA activities in *Cancer pagurus* gills were the same as those determined by^40^ and lower than those observed in SW *Carcinus maenas*, further supporting the general observation that NKA activities are lower in stenohaline vs euryhaline species^29^. Nonetheless, the ability of *Cancer pagurus* to maintain haemolymph osmolality just above the values in dilute seawater demonstrates some control over extracellular osmolality. Such control is likely to be related to the production and efflux of free amino acids from the tissues during low salinity exposure in order to decrease intracellular osmolality and protect against cell swelling^10,28,52^. Previous studies report that *Cancer pagurus* gills have greater capacities than *Carcinus maenas* gills for dealing with deamination resulting from amino acid mobilisation; for instance, they have greater capacities for the active excretion of NH_3_, and have an increased dependency on the metabolism of amino acids^55,56^. Moreover, osmoconforming species tend to have higher gill permeabilities to water and to ions^56,57^. For example, transepithelial conductances are 5 times higher in the posterior gills of *Cancer pagurus* compared with *Carcinus maenas*^56^. Whilst there is some indication that gill permeabilities do not change in osmoconformers after 2 week’s exposure to low salinity^29^, preliminary results show a thickening of the branchial cuticle in *Cancer pagurus* after 6 months exposure to DW, which may indicate a decrease in gill permeability (R. Poulter, B. Ciotti, C. Hauton, unpublished observations).

**Figure 4:**
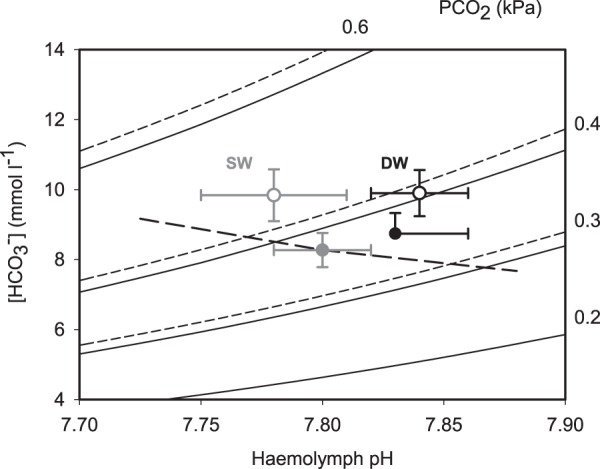
A pH–bicarbonate diagram comparing the main acid-base variables in the haemolymph of *Cancer pagurus* exposed to the 4 treatments: SW = seawater S = 33 (grey symbols); DW = dilute seawater S = 25 (black symbols); CCO_2_ = control *p*CO_2_ (closed circles); HCO_2_ = elevated *p*CO_2_ (open circles). Continuous curved lines represent *p*CO_2_ isopleths for *Cancer pagurus* haemolymph in SW. The associated dotted lines represent the shift in *p*CO_2_ isopleths in DW, resulting from salinity-related changes in dissociation constants for carbonic acid. The broken line represents the non-bicarbonate buffer line (β = 12 mmol l^−1^ pH^−1^) taken from^78^ adjusted for the protein levels measured in *Cancer pagurus* haemolymph. Values given as means ± SEM (*n* = 32 in SW + CCO_2_; *n* = 31 in SW + HCO_2_; *n* = 29 in DW + CCO_2_; *n* = 27 in DW + HCO_2_).

The general accumulation of haemolymph *p*CO_2_ and [HCO_3_^−^] in *Cancer pagurus* over time suggests impairment of CO_2_ excretion, especially after 9 months. This response is difficult to explain but may result from a combination of factors, such as increase in body size reducing CO_2_ excretion rates caused by a fall in weight-specific gill surface area, and/or the limited involvement of ion exchange mechanisms and ion transporting cells in CO_2_ excretion in *Cancer pagurus* gills. Regardless of time, we provide evidence that *p*CO_2_ and [HCO_3_^−^] were consistently higher in crabs exposed to elevated *p*CO_2_ than in controls. Moreover, a key observation in the present study was the diminished disturbance to haemolymph pH in *Cancer pagurus* held in dilute seawater. It is possible that this response was caused by a metabolic alkalosis resulting from an increase in haemolymph [HCO_3_^−^] independently of external changes to offset the increased difference in haemolymph Na^+^ and Cl^−^ concentrations (known as strong ion difference) resulting from exposure to low salinity^58^. An increased deamination of amino acids associated with cell volume control, may also buffer the production of H^+^ via subsequent binding to NH_3_ to give NH_4_^+^ as observed in isolated gill preparations in osmoconforming *Carcinus maenas* on exposure to elevated *p*CO_2_^52^. In addition, increased ammonia excretion via NHE exchangers would increase the loss of H^+^ during low salinity exposure. Although the actual mechanisms causing the metabolic alkalosis remain unclear, the pH-bicarbonate diagram in Fig. 4 illustrates that SW *Cancer pagurus* had lower haemolymph pH than DW crabs and experienced greater disruptions to acid-base status during elevations in seawater *p*CO_2_. Although there was some ability to increase haemolymph [HCO_3_^−^], values were considerably lower (<15 mmol l^−l^) than those reported in osmoregulators during hypercapnia^12^. Moreover, *VATB* was downregulated in low salinity, which may have increased H^+^ availability in the haemolymph supporting the formation of NH_4_^+^, potentially contributing to the metabolic alkalosis. In our study *Cancer pagurus* survived the treatment combinations for 9 months before mortality rates started to increase. *Cancer pagurus* exposed to 50% seawater (S = 15) survived for 15 days by tolerating the salinity-induced changes^31^. This is unlikely to be the case in the present study, because of the time scale involved. Instead, longer-term exposures may allow alterations in the concentration of osmotically active solutes and also changes in gill permeability^28^. The effectiveness of such a strategy, however, was limited ultimately demonstrating the sensitivity of *Cancer pagurus* to combined changes in CO_2_ and salinity.

## Conclusion

Our work emphasises the dominant impact of a relatively understudied climate change variable, salinity, in comparison to elevated *p*CO_2,_ and demonstrates the necessity to study specific salinity/*p*CO_2_ combinations. We also demonstrate that ion transporting capacities are of key importance in terms of predicting responses of marine species to climate change. Mechanisms of active transbranchial ion uptake, which enable euryhaline osmoregulating crabs to invade and exploit estuarine environments have also improved their tolerances to elevated CO_2_. The sustained, salinity-driven increase in gill NKA activities observed in dilute seawater *Carcinus maenas*, as well as the salinity–induced upregulation of 5 out of the 6 genes of interest suggests permanent adjustments in branchial ion exchange to low salinity. Such mechanisms are also sufficient to maintain CO_2_ excretion across the gills at elevated CO_2_, regardless of salinity. The failure to increase branchial ion transporting capacities in the osmoconforming species, *Cancer pagurus*, even in juveniles occupying the low intertidal, shows greater species sensitivity to elevated CO_2_, although there is some respite in dilute seawater. We conclude that osmoconformers with their preferences for stable habitats are poorly equipped for changes in seawater CO_2_ and salinity via the inability to increase ion exchange capacities. Further high resolution studies are urgently required to assess the role of ecological (e.g. behaviour, habitat use etc.) and evolutionary (e.g. genetic diversity) responses in determining whether osmoconformers, such as *Cancer pagurus*, are able to survive a multivariate natural environment. In conclusion, our observations argue for the careful identification of species sensitivity to environmental perturbations and cautions against forecasting potential ecosystem futures based on assessments of community structure at relatively course taxonomic resolution.

## Methods

### Animal collection and maintenance

Juvenile *Carcinus maenas* were collected by hand from low intertidal sites on Anglesey, UK (53°13′48″N, 4°9′W and 53°13′12″N, 4°10′48″W) between March and May 2013 and returned to the Nuffield Laboratory, School of Ocean Sciences, Bangor University within 2 h of collection. *Carcinus maenas* were exposed to treatments starting in June 2013 (body mass = 7.12 ± 0.17 g; carapace width = 30.7 ± 0.2 mm; *n* = 480). Juvenile *Cancer pagurus* from a single panmictic population were collected by hand from Feb to March 2014 at various low intertidal, non-SAC (Special Areas of Conservation) sites from Anglesey (53°24′36″N,4°17′24″W) to South Wales (51°34′12″N,3°58′48″W). Crabs were transported back to the Nuffield Laboratory and returned to seawater within 5 h. *Cancer pagurus* were exposed to treatments from June 2014 (body mass = 21.74 ± 0.74 g; carapace width = 48.3 ± 0.6 mm; *n* = 472). Both species were collected from low intertidal sites characterised by rocky outcrops on generally sheltered shores. Before treatments commenced, crabs were held in fully aerated and recirculated (sand filtered and UV sterilised) seawater at ambient conditions of salinity, *p*CO_2_ (400 µatm), day length and temperature (*Carcinus maenas*: 9.0 ± 0.3 °C, salinity 33.9 ± 0.1, pH_NIST_ 8.03 ± 0.10; *Cancer pagurus*: 12.0 ± 1.6 °C, salinity 34.4 ± 0.1, pH_NIST_ 8.01 ± 0.10) for between one and 4 months. Crabs were fed a food ration of approximately 4% of body mass three times a week; twice on a diet of squid (Squid rings, Bradleys, UK) and once a week with a diet of mussels (*Mytilus edulis*).

### Experimental regime

Juvenile *Carcinus maenas* and *Cancer pagurus* were exposed on separate occasions to elevated *p*CO_2_ to match the ‘business as usual’ scenario for 2100 of ~1000 µatm^59^, and a reduction in salinity to 25 corresponding to the values just below those responsible for initiating osmoregulation in *Carcinus maenas*^29,48^. Crabs were exposed to one of 4 treatments in an aquarium system modified from^60^: ambient *p*CO_2_ (~400 µatm)/seawater (salinity 33); elevated *p*CO_2_ (~1000 µatm)/seawater (salinity 33); ambient *p*CO_2_ (~400 µatm)/dilute seawater (salinity 25); and elevated *p*CO_2_ (~1000 µatm)/dilute seawater (salinity 25). Each treatment consisted of a mixing tank (350 L), a header tank (100 L) and five holding tanks (48 L). Each mixing tank was supplied with natural, filtered (200 µm) and UV sterilized seawater. Seawater dilution was achieved in two of the mixing tanks by adding dechlorinated freshwater vigorously aerated for several hours in an adjoining holding tank (510 L). Salinity was controlled at 25 via conductivity sensors (Eutech Instruments COND 560) calibrated with certified standards (Cole Palmer) every week. Elevated CO_2_ levels were delivered to two of the mixing tanks by controlling the flow of a gas mixture of air and pure CO_2_ via gas line restrictors and flow meters according to^61^. The composition of the air/CO_2_ gas mixture was determined using a Licor LI-840A CO_2_ analyser. The two remaining mixing tanks were fully aerated to represent ambient *p*CO_2_ levels (~400 µatm). Seawater supplied to each header tank from its corresponding mixing tank was gravity fed to five independent holding tanks at a rate of 68 ± 6 L h^−1^ (mean ± SD) and run to waste. The system was housed within a temperature controlled room set at 11 °C and held in a light: dark cycle of 12 L:12D. Further control of seawater temperature in each header tank was achieved by an inline thermostatic heater (Elecro 900 Evo Titanium Digital aquarium Heater, Electro Engineering Ltd., Hertfordshire, UK), offset against a chiller (Aqua Medic TITAN 2000, Aqua Medic Ltd, Coalville, UK). Temperatures were maintained at 11–12 °C across all treatments but allowed to rise in the summer months to 15–16 °C to mimic natural conditions, which is an important consideration during longer-term exposures^62^.

At the start of the experimental exposures, crabs were allocated at random to each of the 25 holding tanks, and progressively exposed to the final treatment combinations over three days. Crabs were held individually in perforated cylindrical plastic containers ranging in size from 0.6 to 3 L with no more than 24 crabs per holding tank. The high flow rate of seawater through the system ensured that the seawater within each holding tank was replaced approximately every 44 min. This minimised any non-treatment effects, such as temperature variation, and maintained the seawater carbonate chemistry at the desired levels in both the holding tanks, and in the individual perforated containers. The latter were unaffected by the biological captivity of the crabs. As the crabs moulted and grew in size, each container was replaced to ensure a constant ratio between crab size and container volume. Crabs were fed 3 times a week as described previously, but left for 48 h without food before sampling.

Crabs were sampled before exposure (baseline) and after 1, 3, 6 months, and 12 months in *Carcinus maenas*, and 1, 3, 6 and 9 months in *Cancer pagurus* (*n* = 6–8 treatment^−1^ month^−1^). Experiments on *Cancer pagurus* were limited to 9 months as mortality rates started to increase and the aim here was to ensure determination of physiological responses to sub-lethal effects. *Carcinus maenas* increased in body mass from 8.63 ± 0.65 (*n* = 26) to 44.16 ± 2.34 (*n* = 24) g, and in CW from 33.1 ± 1.0 (*n* = 26) and 57.3 ± 1.1 (*n* = 24) mm between 1 and 12 months. *Cancer pagurus* increased in body mass from 22.00 ± 2.57 (*n* = 31) to 55.52 ± 5.68 (*n* = 28) g, and in CW from 36.1 ± 2.1 (*n* = 31) to 68.3 ± 1.0 (*n* = 28) mm between 1 and 9 months. In the majority of cases all crabs were in intermoult, but several crabs in the summer sampling months were in early premoult (D_0_ and D_1_). The latter were determined as crabs in which the lower margin of the carapace and the merus of the cheliped remained firm, but the new underlying epidermis was either confluent with the carapace or just beginning to separate at the dorsal anterior edge. No crabs were sampled beyond D_1_ avoiding the physiological changes that are known to occur at D_3_ and D_4_ stages of premoult and the large changes in gene expression reported to occur in postmoult^63^. At each sampling interval, haemolymph samples (300–400 µL) were withdrawn from the infrabranchial sinus with the minimum of disturbance to the crabs and used immediately to determine haemolymph acid-base status and osmolality. Crabs were sacrificed by ablating the thoracic and cerebral ganglia, and gill pair 8 dissected out from the branchial chamber, and flash frozen and stored at −80 °C for the determination of NKA activity (right) and gene expression (left).

### Seawater Chemistry

Daily temperature (°C) and pH_NIST_ were recorded for each treatment using a combined pH electrode and meter (Mettler Toledo SG2 SevenGO, MT Ltd., Leicester, UK), along with daily salinity using a conductivity electrode and meter (Mettler Toledo SG3 SevenGO MT Ltd., Leicester, UK). The pH and conductivity electrodes were calibrated twice weekly with NIST certified pH buffer solutions and standard solutions, respectively. Seawater samples were removed monthly from holding tanks at random for the measurement of Total Alkalinity (A_T_) and Dissolved Inorganic Carbon (DIC), as well as nutrient concentrations (total nitrate, phosphate and silicate). Samples (100 ml) for A_T_ and DIC analysis were taken in triplicate from each of the cylindrical mixing tanks, siphoned into glass containers and preserved with 0.02% mercuric chloride. Carbon chemistry was analysed by the UKOARP Carbonate System Facility at National Oceanographic Centre Southampton. Seawater samples of 60 ml were taken at the same time, filtered (Whatman GFF 0.7 µm), and stored at −20 °C in light proof containers for nutrient analysis at the Scottish Association for Marine Sciences using a Lachat Quikchem 800 Flow Injection Analyser. Carbonate system variables (*p*CO_2_, Ω calcite and Ω aragonite) were calculated with CO2SYS^64^ with refitted constants^65,66^ and are presented in Supplementary Table S1.

### Haemolymph Acid-Base Status and Osmolality

Haemolymph pH was determined by injecting a small subsample (~200 µL) past the face of an E310 glass pH electrode connected to an E351 reference electrode housed in a BC202 blood gas cell (Cameron Instrument Company) and supplied with circulating water at the appropriate temperature. The electrodes were connected to a pH/blood gas meter (Radiometer PHM73) and calibrated using NIST analytical buffers at regular intervals (7.45 and 8.06 at 15 °C, Fluka Analytical). Total CO_2_ (TCO_2_) was determined according to the Cameron technique^67^. Small subsamples (40 µL) were injected into a 2 mL chamber filled with 0.01 N HCl and maintained at 38 °C. A CO_2_ electrode (Radiometer E5037) in contact with the chamber was connected to a pH/blood gas meter (Radiometer PHM73) and calibrated with standard solutions of NaHCO_3_ (10, 20 and 40 mmol L^−1^). A further haemolymph subsample of 15 µL was used to determine osmolality using a freezing point osmometer calibrated with deionised water and a standard solution (Osmomat 030, Gonotec GmbH, Berlin, Germany). Partial pressure of CO_2_ (*p*CO_2_) and HCO_3_^−^ concentrations were calculated from measured values of haemolymph pH and TCO_2_ using the Henderson-Hasselbach equation. The apparent first dissociation constant for carbonic acid (pK’_1_) and the solubility coefficients for CO_2_ (αCO_2_) were adjusted for salinity and temperature provided by^68^.

### Branchial Na^+^/K^+^-ATPase (NKA) activities

NKA activities were determined in gill 8 homogenates by microassay^69^, in which the hydrolysis of ATP is enzymatically linked to the oxidation of NADH. Protein concentrations in the gill homogenates were determined using the micro-modification of the Pierce BCA Protein Assay (Thermo Scientific). NKA activity was expressed as µmol ADP produced mg^−1^ protein h^−1^.

### Gene Expression

The genes of interest were chosen for their role in the exchange of ions and acid-base equivalents across the gill epithelia of aquatic crabs. Na^+^/K^+^-ATPase on the basolateral membrane drives Na^+^ uptake from the surrounding seawater via an apical Na^+^/K^+^/2Cl^−^ symporter and possibly through the apical 2Na^+^/H^+^ antiporter (NHE). Cytoplasmic carbonic anhydrase converts CO_2_ into the acid-base equivalents HCO_3_^−^ and H^+^ for use as counterions for Cl^−^ or Na^+^ uptake, via anion and NHE exchangers, respectively. A basolateral location for NHE suggests a role in the uptake of HCO_3_^−^ across the gill epithelia as speculated by^52^. The gpi-linked carbonic anhydrase located on the basolateral membrane dehydrates HCO_3_^−^ and H^+^ into CO_2_ for diffusion across the gill epithelium into seawater. These mechanisms are based on models summarised by^10,29,52,70^.

Expression of selected gene targets was measured in gill 8 using a relative quantification strategy by real-time PCR with SYBR Green I detection chemistry following MIQE guidelines^71^. Gill tissue (<0.1 g) was homogenized in 1 ml TRI reagent (Sigma-Aldrich, Dorset, UK) for 4 min on a TissueLyserII (QIAGEN, Manchester, UK) and extracted according to the manufacturer’s protocol to yield high purity, high integrity total RNA, as confirmed by Nanodrop^TM^ (mean A_260/280_ = 1.9; Thermo Fisher Scientific, Leics, UK), and an Experion^TM^ RNA StdSens Analysis kit (mean RQI > 9.0; Bio-Rad, Herts, UK). Total RNA samples were treated with RQ1 RNase-free DNase (Promega, Hants, UK) and reverse-transcribed from poly dT_20_ primers using SuperscriptIII^TM^ (LifeTechnologies, Strathclyde, UK). We used PrecisionPLUS Mastermix (Primer Design, Hampshire, UK) for *Carcinus maenas* and iQ SYBR® Green Supermix (Bio-Rad, Herts, UK) for *Cancer pagurus*. Primers were designed using PrimerExpress^TM^ (Applied Biosystems®, California, USA) or Beacon Designer^TM^ (Premier Biosoft, CA, USA) against target DNA sequences obtained from GenBank or through degenerate PCR on conserved regions identified through Clustal Omega protein alignments^72^. Details of gene targets, primers and assay performance are provided in Supplementary Table S2. Technical duplicates were run on a Corbett Rotorgene 3000 (QIAGEN, Manchester, UK) with the following reaction conditions: 1 cycle of 95 °C for 10 min (*Carcinus maenas*) or 3 min (*Cancer pagurus*); 40 cycles of 95 °C for 10 s (*Carcinus maenas*) or 15 s (*Cancer pagurus*) and 60 °C for 60 s, 1 cycle of 72 °C for 45 s and a final ramp to 95 °C of 1 °C per 5 s. Reaction specificity was confirmed in all reactions by inspecting melt curves.

Threshold cycles for all target genes were calibrated across runs using three inter-run calibrators and normalized to the best combination of five candidate reference genes (*act, AK, eefl1A, gapdh, tub*) using GeNorm™^73^ and qBase+™ (Biogazelle, Zwijnaarde, Belgium) to establish Calibrated Normalized Relative Quantities (‘CNRQs’)^74^. The logarithm (base 10) of CNRQs were used in statistical analyses because variation in gene expression follows a log normal distribution^75,76^. Differences in log(CNRQ) are equivalent to log fold changes.

### Statistical analysis

Means for all seawater parameters were compared among all 4 treatments, after testing for normality using the Kolmogorov-Smirnov test and homogeneity of variance using the Levene Statistic. For parametric data, we used a one-way ANOVA and a Student-Newman-Keuls (SNK) *post hoc* test to make pairwise comparisons between treatments. For those comparisons that did not meet the assumptions for normality, we used a Kruskal-Wallis test, and then Dunn’s post hoc test with Bonferroni correction for pairwise comparisons (SPSS version 22, Chicago).

The influence of CO_2_, salinity, sampling month (all fixed factors) and body size (covariate) on the response variables (haemolymph acid-base variables, osmolality, branchial NKA activities and gene expression) were tested using a generalised least squares (GLS) approach. In our full model, we fully crossed all explanatory variables and allowed heterogeneity in variance among each CO_2_, salinity and sampling time combination (Tables 1 and 2). We then identified the best variance structure by comparing candidate models having reduced variance structures using the gls function in R (version 3.3.2). The best candidate model was selected as that with the lowest Akaike’s Information Criterion with small sample correction (AICc). Once the best variance structure had been established, we attempted to remove fixed effects from the global model after refitting with maximum likelihood (ML) and the appropriate variance structure. Terms were removed if this did not increase the AICc. In cases where dropping a term increased the AICc by less than two, the decision to drop terms was made on the basis of log-likelihood ratio tests (α = 0.05) (Supplementary Tables S3–S8). All analyses were performed using the *nlme* package in R (version 3.3.2)^77^. When response variables were influenced by independent factors, the SNK *post hoc* test was used to determine significant differences among multiple means (SPSS, version 22). To aid interpretation, we report the simpler models for both species in Results, and outline model selection in Supplementary Tables S3–S8.

Exposing crabs for many months introduces confounding factors, such as increases in body size, and in this study, seasonal changes in temperature. To account for changes in body size, we included carapace width (CW) as a covariate in our statistical models. CW was chosen as a proxy for body size to avoid inaccuracies caused by water retention in the branchial chambers of the crabs during weighing. Throughout there was some potential level of co-variation in time and body size, but not all time intervals were confounded by size differences. Throughout exposure, small/damped seasonal changes in temperature occurred, but changes were similar across all treatments and there was no tank effect as variation among tanks was smaller than the variation within tanks.

## Electronic supplementary material

Supplementary Information

## Data Availability

Data pertaining to the manuscript will be made available via the Research Information Management System PURE.
