# Supplementary material for: Sensitivity to near-future CO2 conditions in marine crabs depends on their compensatory capacities for salinity change
Source: Sci Rep. 2018 Oct 23;8:15639. doi: 10.1038/s41598-018-34089-0 (PMC6199311; doi:10.1038/s41598-018-34089-0)
Supplement: Supplementary file 1 — Supplementary Information [file 41598_2018_34089_MOESM1_ESM.pdf]

Sensitivity to near-future CO_2 conditions in marine crabs depends on their compensatory capacities for salinity change

Nia M. Whiteley, Coleen C. Suckling, Benjamin J. Ciotti, James Brown, Ian D. McCarthy, Luis Gimenez and Chris Hauton

Table S1. Measured and estimated seawater treatment parameters for both species.

Species	Parameter	SW + CCO_2 (control)	SW + HCO_2	DW + CCO_2	DW + HCO_2
Carcinus maenas	**pH_{NIST}**	**8.03 ± 0.01[a]**	**7.72 ± 0.01[b]**	**8.09 ± 0.01[c]**	**7.78 ± 0.01[d]**
	**Salinity**	**33.22 ± 0.04[a]**	**33.17 ± 0.05[a]**	**25.47 ± 0.09[b]**	**25.39 ± 0.07[b]**
	**Temp (˚C)**	**12.53 ± 0.06[a]**	**12.71 ± 0.06[a]**	**12.58 ± 0.05[a]**	**12.64 ± 0.06[a]**
	**A_T (μmol kg^{-1})**	**2,272 ± 4[a]**	**2,272 ± 4[a]**	**1,813 ± 5[b]**	**1,825 ± 5[b]**
	**DIC (μmol kg^{-1})**	**2,120 ± 4[a]**	**2,222 ± 5[b]**	**1,716 ± 7[c]**	**1,795 ± 4[d]**
	pCO_2 (μatm)	527 ± 13[a]	1125 ± 56[b]	508 ± 49[a]	930 ± 27[b]
	Ω calcite	2.80 ± 0.06[a]	1.54 ± 0.05[b]	1.88 ± 0.07[b]	1.06 ± 0.03[c]
	Ω aragonite	1.78 ± 0.04[a]	0.98 ± 0.03[b]	1.16 ± 0.05[b]	0.66 ± 0.02[c]
Cancer pagurus	**pH_{NIST}**	**7.99 ± 0.01[a]**	**7.67 ± 0.01[b]**	**8.03 ± 0.001[c]**	**7.77 ±0.01[d]**
	**Salinity**	**33.17 ± 0.05[a]**	**33.27 ± 0.05[a]**	**25.60 ± 0.09[b]**	**25.32 ± 0.08[b]**
	**Temp (˚C)**	**12.66 ± 0.09[a]**	**12.78 ± 0.09[a]**	**12.75 ± 0.08[a]**	**12.68 ± 0.09[a]**
	**A_T (μmol kg^{-1})**	**2,303 ± 16[a]**	**2,297 ± 10[a]**	**1,821 ± 13[b]**	**1,826 ± 6[b]**
	**DIC (μmol kg^{-1})**	**2,143 ± 8[a]**	**2,243 ± 10[b]**	**1,721 ± 13[c]**	**1,787 ± 9[d]**
	pCO_2 (μatm)	570 ± 45[a]	1138 ± 77[b]	481 ± 30[a]	874 ± 56[b]
	Ω calcite	3.01 ± 0.28[a]	1.61 ± 0.08[b]	1.91 ± 0.08[b]	1.17 ± 0.05[c]
	Ω aragonite	1.92 ± 0.18[a]	1.03 ± 0.05[b]	1.19 ± 0.05[b]	0.73 ± 0.03[c]

A_T = total alkalinity, DIC = dissolved inorganic carbon, SW = full strength seawater, DW = diluted seawater, CCO_2 = ambient pCO_2, HCO_2 = elevated pCO_2.

Values are means ±SEM.

Data represent daily values for pH, salinity and temperature and monthly values for remaining parameters.

Measured values shown in bold used to calculate pCO_2, calcite and aragonite saturation states (Ω calcite and Ω aragonite).

Different letters indicate significant differences among treatments (P <0.05) using one-way ANOVA or Kruskal-Wallis test, and appropriate *post hoc* tests.

Table S2. Details of gene targets, primers and assay performance for real-time PCR analyses.

Target	Genbank accession no.	F primer	R primer	Amplicon size (bases)	F / R primer conc. (µM)	Efficiency	Linear range (µg in 50µL)
Carcinus maenas							
act	MG554415	TCACCAACTGGGACGACATG	GCCACACGGAGCTCATTGTAG	64	0.3 / 0.3	1.009	0.4 - 0.0002
AK	AF167313.1	CCAATGCTTGCCGCTACTG	AGGAAGGTCTTGTTGTCGTTGTG	64	0.3 / 0.3	0.976	0.4 - 0.0002
AE	CX994129	CCTGCTTGTGTGCGAACAGT	GATTCACCAGGTGCATGTGTAGA	74	0.3 / 0.3	0.995	0.2 - 0.0002
CAc	EU273943.1	CATAAAGGGCAGGCGATCAG	ACCGTTCTTGGGTAGGAAGGTT	66	0.3 / 0.3	1.040	0.2 - 0.002
gpi-CA	EU273944.1	CACGCCTACCTGCAACGAA	TGACGCTCGGAGATGGAGAT	69	0.3 / 0.3	1.059	0.2 - 0.0002
eef1A	MG430409	TCTGGATGGAGGCTCAATGTT	AGAGCGGCAGCTATGAGTTCA	65	0.3 / 0.3	0.924	0.4 - 0.00002
gapdh	MG430408	TGCGGTGTGAATTTGGAGAA	GGTGCAGGAGGCATTGGAT	63	0.3 / 0.3	1.007	0.4 - 0.0002
NKAα	AY035550.1	GAGGGATGGAAGGCTCTCTCTAG	ACGTCATCTTGGGCAGTCTTG	74	0.3 / 0.3	1.087	0.4 - 0.002
NHE	U09274.1	GCGCCTCCACCTACAAGAGA	TGGCCGATATCCGATTGTG	67	0.3 / 0.3	1.129	0.2 - 0.0002
tub	MG430410	CTCTGCTTCTTTGCGGATAACA	CACTACACGGAAGGTGCTGAAC	63	0.3 / 0.3	0.972	0.4 - 0.0002
VATB	AF189779.2	CATGATGACCACAAGGATAACTTTG	CCTGGCAGTCTCCATGTTGA	69	0.3 / 0.3	0.973	0.4 - 0.002
Cancer pagurus							
act	FR687021.1	GTGACATCAAGGAAAAGCTGTGTT	GGAAGCGGCAGTGGTCAT	68	0.05 / 0.05	0.933	0.4 - 0.000014
AK	**MF972215**	GATGACCACTTCCTCTTC	GTCGTTGTTGTGGTAGAT	99	0.3 / 0.3	0.966	0.4 - 0.000014
AE	**MF972216**	CTGAGCACAAGGGAAATACTCATTT	CCTCTCCCACCATTATGTTGGA	78	0.3 / 0.05	1.012	0.4 - 0.0014
CAc	**MF972217**	ATGGCGAGACCCTTGTGACT	GAACACCCGGAGATGAAAAAGA	65	0.3 / 0.3	0.905	0.4 - 0.0014
gpi-CA	**MF972218**	TTTGGCAGTACTTGGTGTGATGT	AGGAGCAAGAGCCGGGTTA	61	0.3 / 0.3	0.972	0.4 - 0.00004
eef1A	**MF972219**	AACCACCCTGGTCAGATCCA	TGCAGGCAATATGTGCAGTGT	70	0.3 / 0.3	0.910	0.4 - 0.00014
gapdh	**MF972220**	TCCCCGAGTTGAATGGAAAG	AGATCCACCACGGACACATCA	73	0.05 / 0.05	0.984	0.4 - 0.00014
NKAα	**MF972221**	CCAGAGACTCAACATTCC	GTCATCCAGTTGTTCAGA	106	0.3 / 0.3	1.005	0.4 - 0.00014

tub	**MF972222**	CCCTACCCCAGAATC CACTTC	CATGGTAAGCCTTCTC AGCAGAA	70	0.05 / 0.05	0.968	0.4 - 0.000004
VATB	**MF972223**	CTGACTATGCCTAAT GATGA	TAGATTTGACGGTTGT GAA	107	0.3 / 0.3	0.982	0.4 - 0.0014

Abbreviations: actin, *act*; arginine kinase, *AK*; anion exchange protein, *AE*; cytoplasmic carbonic anhydrase, *CAc*; glycosyl-phosphatidylinositol-linked carbonic anhydrase, *gpi-CA*; elongation factor 1 A, *eef1A*; glyceraldehyde-3-phosphate dehydrogenase, *gapdh*; Na^+/K^+ ATPase alpha subunit, *NKAα*; Na^+/H^+ exchanger, *NHE*; tubulin, *tub*; and vacuolar-type H^+ ATPase subunit B, *VATB*.

New Accession numbers in bold represent targets sequenced for this study.

Linear range refers to the concentration of the original mRNA sample in the final 50 µL real-time PCR reaction mix.

Table S3. Model selection for the physiological variables in *Carcinus maenas*.

Full term	Term removed	pH AICc	pCO_2 AICc	$[HCO_3^-]$ AICc	Osmolality AICc	NKA AICc
4-way factorial		-189.77	324.2	602.1	1237.4	298.0
3-way factorial		-208.65	312.9	590.9	1228.5	288.8
3-way terms with CW		-200.97	302.2	580.1	1221.9	282.1
	CW:pCO_2:Time	-218.06	292.7	570.1	1213.1	277.5
	CW:Sal:Time	-217.71	293.5	572.6	1216.7	274.4
	CW:Sal:pCO_2	-209.37	299.4	578.0	1218.4	278.4
2-way factorial		-224.29	283.1	563.2	1209.7	266.3
	pCO_2:Time	-231.47	274.7	554.3	1201.2	258.0
	Sal:pCO_2	-224.47	280.7	562.0	1206.9	263.2
	Sal:Time	-232.55	275.2	556.1	1207.9	263.2
2-way with CW		-240.13	265.3	546.2	1197.5	253.5
	CW:Time	-247.10	260.0	541.9	1192.1	248.6
	CW:Sal	-242.45	263.0	543.7	1197.3	250.9
	CW:pCO_2	-241.95	266.4	545.7	1194.9	250.9
Additive		-250.80	258.0	538.5	1189.8	243.9
	Sal	-250.00	256.2	536.2	**1221.8**	**298.8**
	pCO_2	-252.40	256.0	536.2	1187.4	244.7
	Time	**-248.60**	**261.6**	**546.5**	**1196.8**	241.5
	CW	-252.90	256.0	536.7	1188.1	243.8

Abbreviations: CW = carapace width; Sal = salinity; pCO_2 = partial pressure of CO_2; NKA = Na^+/K^+-ATPase activity.

Models selected using generalised linear square models and Akaike Information Criteria (AIC_c) for all dependent variables (haemolymph acid-base status and osmolality, and gill NKA activities). Terms in bold are retained in the models.

Table S4. Likelihood ratio tests (LR tests) for the selected models in *C. maenas*.

Variable	Term	AICc	LR test	*P*
Haemolymph pH	Time	-248.60	9.09	0.028
Haemolymph pCO_2	Time	261.6	10.50	0.015
Haemolymph $[HCO_3^-]$	Time	546.5	14.92	0.002
Haemolymph osmolality	Salinity	1221.8	13.96	0.003
	Time	1196.8	34.41	<0.001
Branchial NKA activity	Salinity	298.8	57.28	<0.001

P values correspond to comparison of full term (S3) and a reduced model lacking the respective term.

Table S5. Model selection for physiological variables in *Cancer pagurus.*

Full term	Term removed	pH AICc	pCO_2 AICc	[HCO_3^-] AICc	Osmolality AICc	NKA AICc
4-way factorial:		-106.0	448.8	632	1386.5	107.1
3-way factorial		-116.3	437.2	622	1386.5	102.8
3-way terms with CW		-126.6	433.7	616	1382.0	95.6
	CW:pCO_2:Time	-133.7	425.6	607	1375.5	96.9
	CW:Sal:Time	-131.4	424.6	607	1375.8	86.1
	CW:Sal:pCO_2	-130.1	431.5	615	1378.6	92.8
2-way factorial		-141.7	415.7	599	1366.6	85.1
	pCO_2:Time	-148.7	408.2	591	1361.2	77.8
	Sal:pCO_2	-144.7	413.2	596	1365.7	82.2
	Sal:Time	-149.2	411.5	595	1361.0	77.2
2-way with CW		-158.3	402.8	586	1355.4	68.0
	CW:Time	-165.3	396.0	579	**1357.7**	65.5
	CW:Sal	-160.6	400.2	584	1352.9	65.5
	CW:pCO_2	-160.3	401.7	584	**1357.8**	67.5
	Sal				**1511.0**	
Additive		169.4	392.5	577	1358.3	62.5
	Sal	**-164.0**	390.3	575	1503.3	60.2
	pCO_2	-170.5	**396.3**	**583**	*1361.0*	60.7
	Time	**-143.5**	**420.4**	**599**	*1361.4*	59.6
	CW	-170.3	**403.8**	**582**	-	**67.5**

Abbreviations: CW = carapace width; Sal = salinity; pCO_2 = partial pressure of CO_2; NKA = Na^+/K^+-ATPase.

Models selected using generalised linear square models and Akaike Information Criteria (AIC_c) for all dependent variables (haemolymph acid-base status and osmolality, and gill NKA activities). Terms in bold are retained in the models.

Table S6. Likelihood ratio tests (LR test) for the selected models in *C. pagurus*.

Variable	Term	AICc	LR test	*P*
Haemolymph pH	Salinity	-164.0	7.90	0.005
	Time	-143.5	33.07	<0.001
Haemolymph pCO_2	pCO_2	396.3	6.23	0.013
	Time	420.4	35.06	<0.001
	CW	403.8	13.78	<0.001
Haemolymph [HCO_3^-]	pCO_2	583.0	9.06	0.003
	Time	599.0	28.98	<0.001
	CW	582.0	7.64	0.006
Haemolymph osmolality	CW:pCO_2	1357.8	4.90	0.027
	CW:Time	1357.7	9.69	0.021
	Salinity	1511.0	160.50	<0.001
Branchial NKA activity	CW	67.5	7.371	0.007

P values correspond to comparison of full term (S5) and a reduced model lacking the respective term.

.

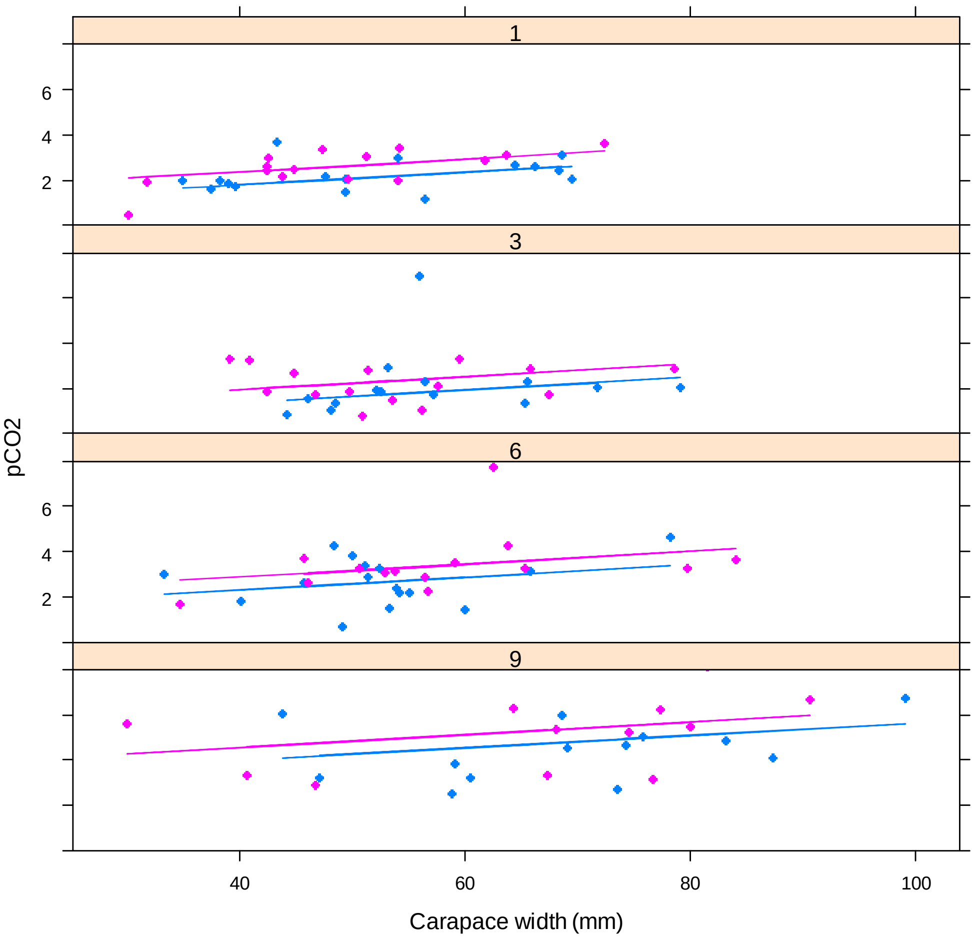

Figure S1. Relationship between body size expressed as carapace width and haemolymph pCO_2 (kPa) for individual *Cancer pagurus* exposed to control pCO_2 (blue lines and symbols) and elevated pCO_2 (pink line and symbols) at different sampling times (1, 3, 6 and 9 months). Lines represent predicted values, and individual data points are observed values. The model parameters suggest a consistent effect of CO_2 levels irrespective of the effect of time and body size.

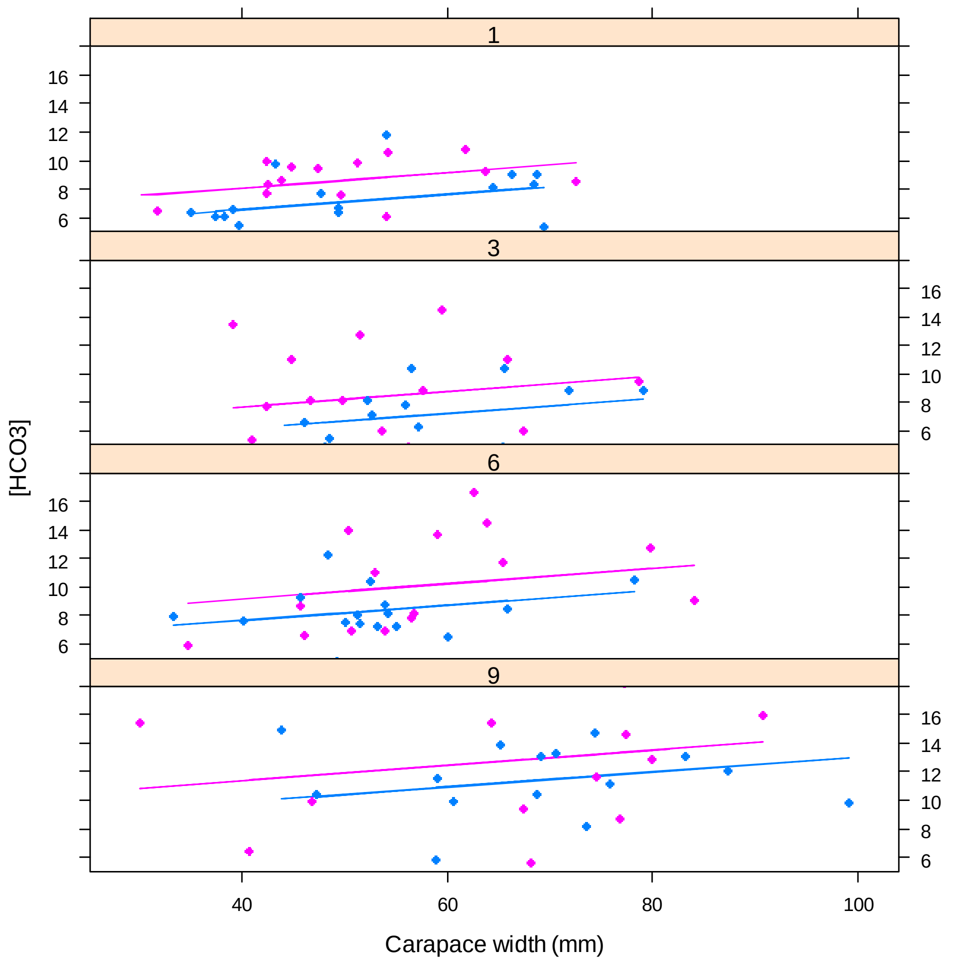

Figure S2. Relationship between body size expressed as carapace width and haemolymph [HCO_3^-] (mmol l^{-1}) for individual *Cancer pagurus* exposed to control *p*CO_2 (blue lines and symbols) and elevated *p*CO_2 (pink line and symbols) at different sampling times (1, 3, 6 and 9 months). Lines represent predicted values, and individual data points are observed values. The model parameters suggest a consistent effect of CO_2 levels irrespective of the effect of time and body size.

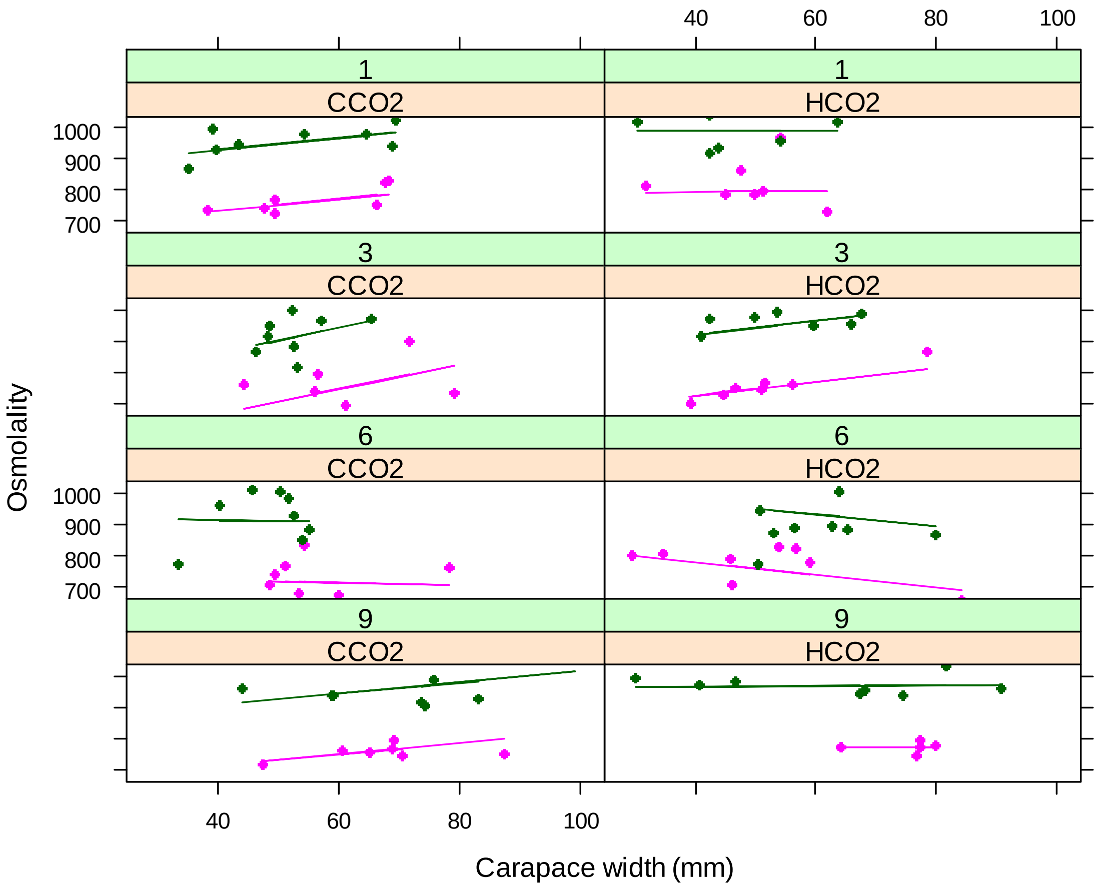

Figure S3. Observed and predicted effects of body size (carapace width), CO_2 (C= control pCO_2, H= elevated pCO_2), sampling times (1, 3, 6 and 9 months) and salinity (green line and symbols = full strength SW; pink lines and symbols = DW) on haemolymph osmolality (mosmol kg^{-1}) in *Cancer pagurus*. Predictions are based on parameter estimates.

Table S7. AICc values and outcomes from model selection for gene expression (log(CNRQ)) in *Carcinus maenas*.

Full model	Term removed	*AE* AICc	*CAc* AICc	*gpi-CA* AICc	*NKAα* AICc	*NHE* AICc	*VATB* AICc
4-way factorial		87.90	240.9	191.3	120.24	159.6	102.19
	CW:Sal:pCO$_2$:Time	80.00	246.2[c]	186.7	110.32	151.1	95.05
3-way factorial		80.00	246.2	186.7	110.32	151.1	95.05
	Sal:pCO$_2$:Time	78.33	243.0	180.0	101.05	150.3	85.85
3-way terms with CW		78.33	243.0	180.0	101.05	150.3	85.85
	CW:pCO$_2$:Time	69.58	**248.6**	170.8	96.54	150.9[b,c]	78.46
	CW:Sal:Time	70.99	**245.9**	171.1	99.64	146.7	78.35
	CW:Sal:pCO$_2$	75.95	240.5	178.3	102.22[b,c]	147.3	84.42
2-way factorial		62.13	*245.1*	162.8	95.68	145.8	71.61
	pCO$_2$:Time	55.54	*238.6*	156.0	89.14	139.7	63.87
	Sal:pCO$_2$	62.76[a]	*243.0*	160.5	96.07[a]	145.0	69.44
	Sal:Time	55.00	*237.4*	157.4	89.94	140.0	70.29
2-way with CW		50.41	*229.5*	149.2	84.16	134.4	61.46
	CW:Time	**56.21**	*224.2*	148.4	79.25	**142.4**	58.62
	CW:Sal	48.03	*227.6*	146.7	81.77	**137.3**	59.02
	CW:pCO$_2$	47.86	*227.1*	147.6	86.64[d]	134.4	61.62[a]
Additive		52.68	*219.9*	146.0	77.70	143.4	55.87
	Sal	51.44	*264.0*	**151.0**	**94.95**	*146.2*	**62.66**
	pCO$_2$	50.94	*217.6*	143.8	78.14[a]	142.9	53.72
	Time	*51.21*	*221.2*	140.5	74.87	*140.1*	52.80
	CW	*53.72*	*220.2*	144.6	78.27[a]	*155.3*	53.63

Further investigation of the higher order interactions for *CAc*, *NKA*α and *NHE* were conducted to test for biological relevance via individual plots of all factor combinations, and by simplifying the models according to AICc criteria and LR tests.

a Removed because log-likelihood ratio test not significant (alpha = 0.05)

b Log-likelihood ratio test significant (alpha = 0.05)

c interaction removed to explore simpler models

d Could be removed from the best model (AICc for Additive + CW: pCO$_2$ = 76.94; AICc for Additive = 77.70, LLR test not significant (alpha = 0.05)).

Bold text: terms retained in the final model.

Grey text & italics: terms that cannot be tested due to higher order interactions

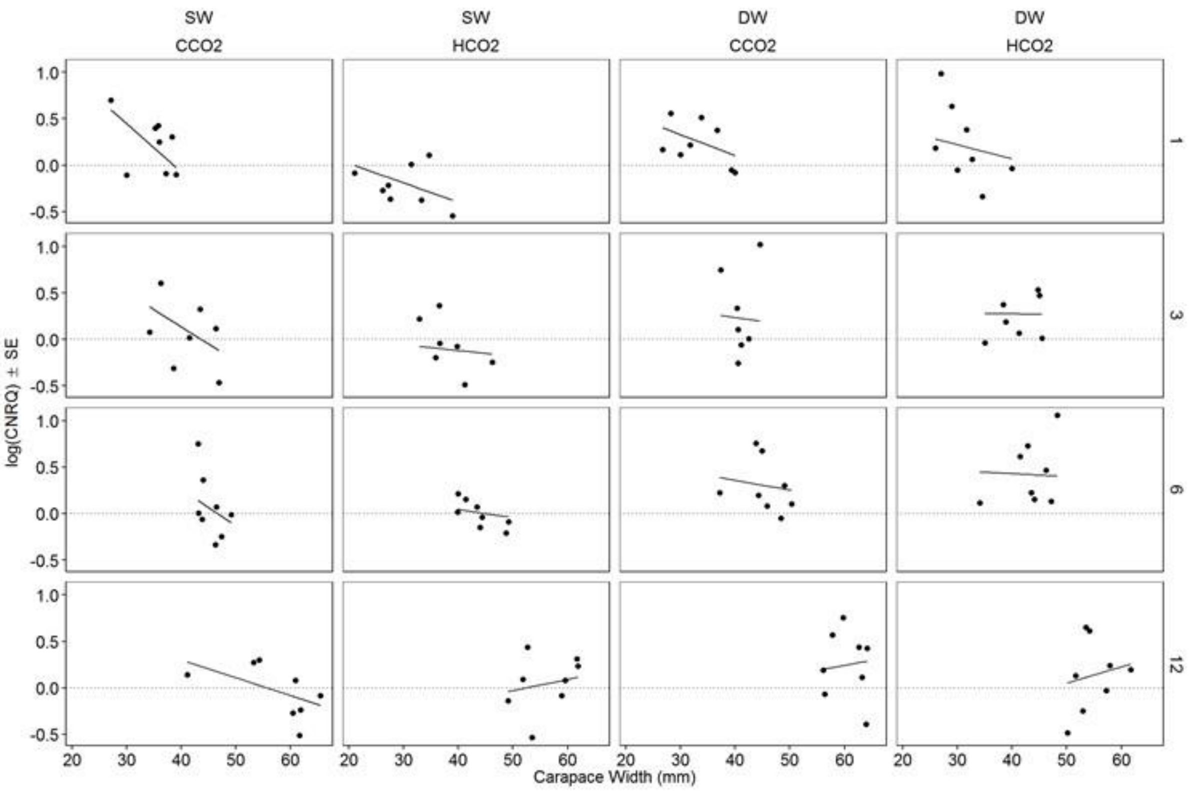

Figure S4. Relationship between *NKAα* expression in the posterior gills of *Carcinus maenas* and body size given as carapace width in all 4 factor combinations: SW/control pCO_2; SW/ elevated pCO_2; DW/control pCO_2; DW/elevated pCO_2, where SW = seawater at a salinity of 33 and DW = dilute seawater at a salinity of 25. Gene expression shown as differences in log(CNRQ). Straight lines constructed from parameter estimates. Horizontal broken line represents the mean for baseline crabs. Overall, figure demonstrates that although the CW:Sal:pCO_2 term was significant (Table S7), the relationship between NKAα expression and carapace width is inconsistent among salinity and pCO_2 treatments: simpler models were therefore explored. SW = seawater at a salinity of 33; DW = dilute seawater at a salinity of 25; CCO_2 = ambient pCO_2; HCO_2 = elevated pCO_2.

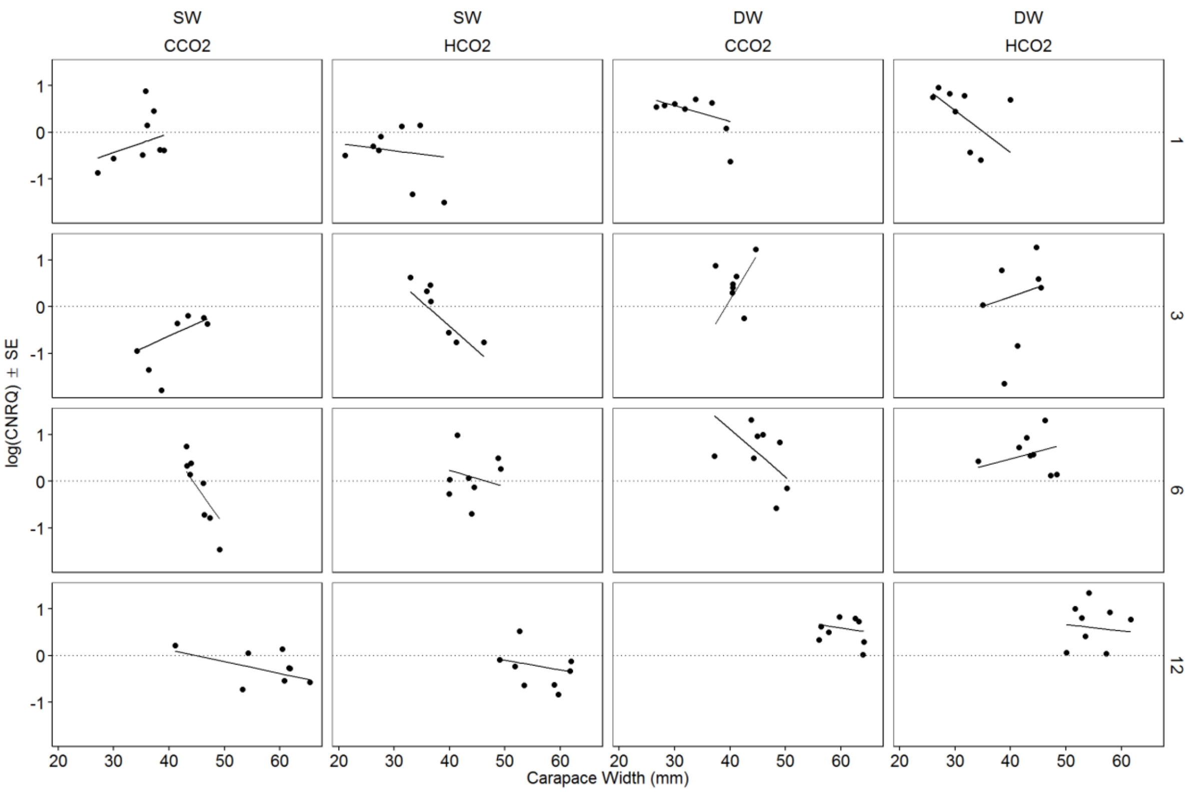

Figure S5. Relationship between *CAc* expression in the posterior gills of *Carcinus maenas* and body size given as carapace width in all 4 factor combinations: SW/control pCO_2; SW/ elevated pCO_2; DW/control pCO_2; DW/elevated pCO_2, where SW = seawater at a salinity of 33 and DW = dilute seawater at a salinity of 25. Gene expression shown as differences in log(CNRQ). Log(CNRQ) is plotted relative to the mean for baseline crabs (horizontal broken lines). Straight, solid lines represent fits of the best model (Table 2).

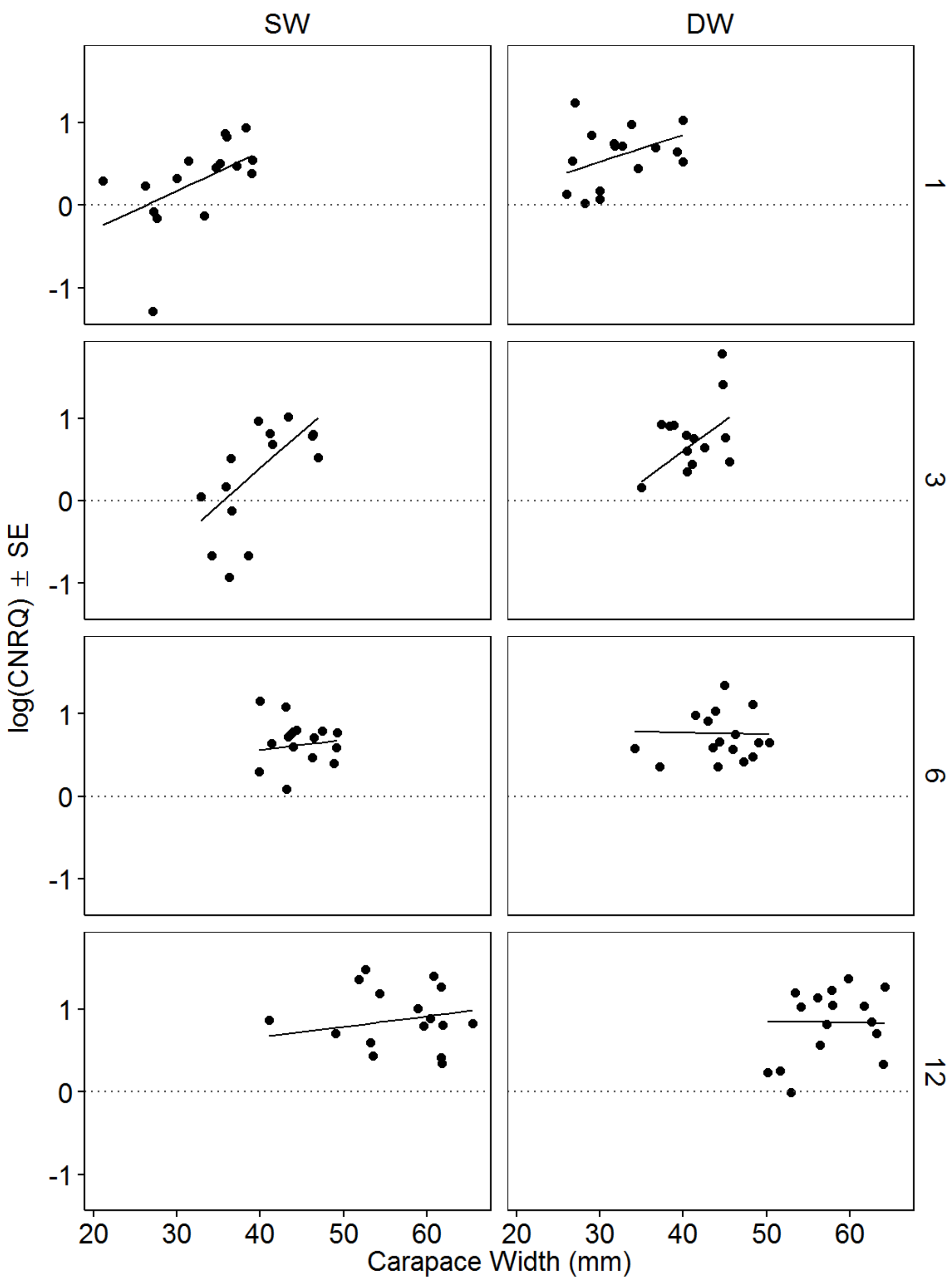

Figure S6. Two-way interactions between body size and time, and body size and salinity for *NHE* expression in the posterior gills of *Carcinus maenas.* SW = control salinity at 33, and DW = dilute seawater at 25. Time given as 1, 3, 6 and 12 sampling months. Log(CNRQ) is plotted relative to the mean for baseline crabs (horizontal broken lines). Straight, solid lines represent fits of the best model (Table 2). Overall, body size had more of an effect on *NHE* expression at one and 3 months, with a decrease in *NHE* expression in smaller crabs in DW.

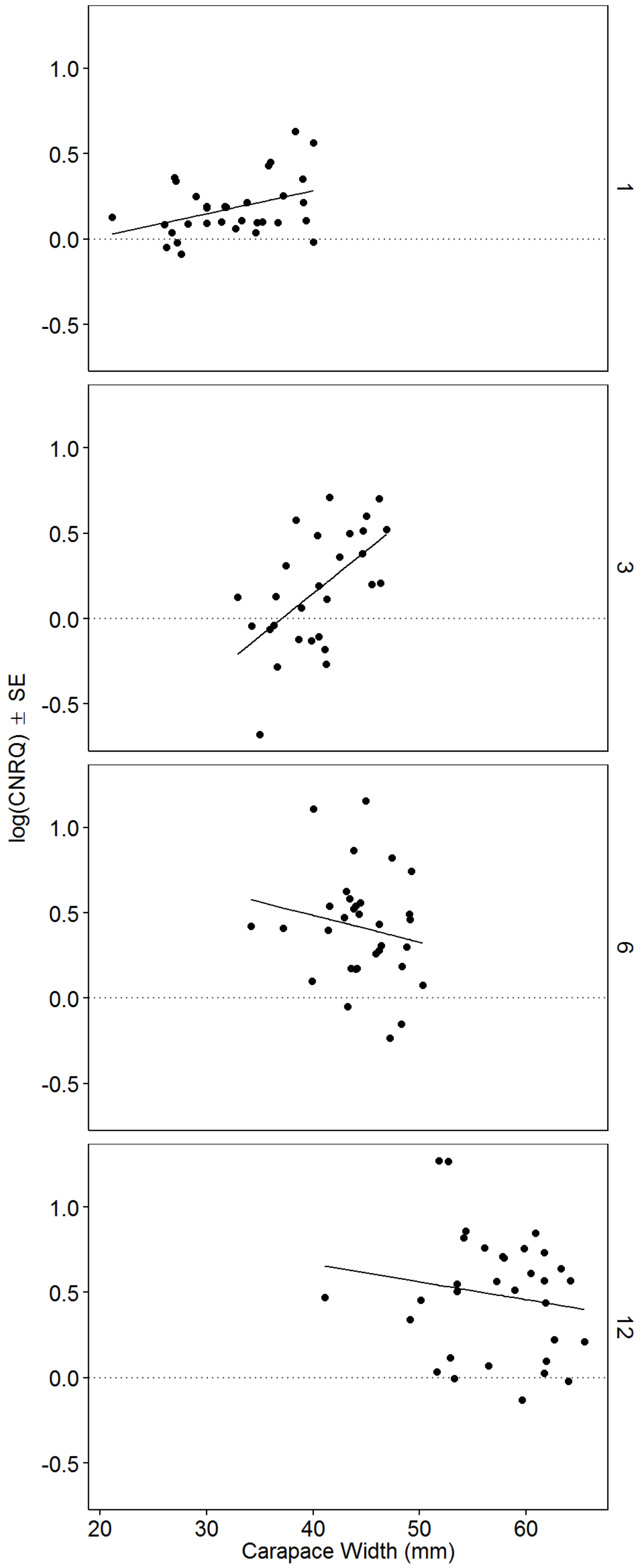

Figure S7. Relationship between *AE* expression in the posterior gills of *Carcinus maenas* and body size given as carapace width and time as 1, 3, 6 and 12 months. Log(CNRQ) is plotted relative to the mean for baseline crabs (horizontal broken lines). Straight, solid lines represent fits of the best model (Table 2).

Table S8. AICc values and outcomes from model selection for gene expression (log (CNRQ)) in *Cancer pagurus*.

Full model	Term removed	*AE* AICc	*CAc* AICc	*gpi-CA* AICc	*NKAα* AICc	*NHE* AICc	*VATB* AICc
4-way factorial		305.0	109.13	128.26	81.15	na	67.96
	CW:Sal:pCO_2:Time	297.8	99.13	123.96	82.54[b,c]	na	61.16
3-way factorial		297.8	99.13	123.96	82.54	na	61.16
	Sal:pCO_2:Time	293.3	90.15	118.51	75.02	na	51.40
3-way terms with CW		293.3	90.15	118.51	75.02	na	51.40
	CW:pCO_2:Time	284.1	83.51	109.28	67.40	na	43.05
	CW:Sal:Time	284.4	81.42	110.71	74.12	na	44.93
	CW:Sal:pCO_2	291.6	87.15	118.61[a]	73.66	na	48.30
2-way factorial		274.3	73.71	101.92	64.21	na	34.32
	pCO_2:Time	266.5	66.44	93.98	56.83	na	27.82
	Sal:pCO_2	271.5	71.80	99.14	61.66	na	32.33
	Sal:Time	267.2	68.34	94.07	58.52	na	26.61
2-way with CW		257.1	59.98	83.96	49.23	na	18.54
	CW:Time	251.9	53.51	80.96	44.17	na	13.10
	CW:Sal	255.8	57.50	82.68	48.11	na	16.93
	CW:pCO_2	254.8	57.48	81.60	46.74	na	16.09
Additive		248.0	48.91	76.88	41.41	na	9.02
	Sal	245.7	47.12	75.10	39.15	na	**16.49**
	pCO_2	249.6[b,d]	46.82	75.86	39.18	na	7.53
	Time	245.3	**55.62**	70.92	34.71	na	**20.32**
	CW	248.6[a]	46.71	77.50[a]	41.33	na	**14.19**

The fourth order interaction was significant for *NKA*α although the resulting increases in AICc were relatively small (<2). No terms were retained in the model after removal of this 4-way interaction. In all cases, additional simplification resulted in models with consistently lower AICc values than the complex models.

a Removed because log-likelihood ratio test not significant (alpha = 0.05)

b Log-likelihood ratio test significant (alpha = 0.05)

c interaction removed to explore simpler models

d Could be removed from the best model (AICc for pCO_2 only model = 247.0; AICc without pCO_2 = 247.7, LR test not significant (alpha = 0.05)).

Bold text: terms retained in the final model
